# Influence of Moisture Invasion on the Deterioration of Epoxy Resin Performance, and Modification and Enhancement Methods

**DOI:** 10.3390/ma18184243

**Published:** 2025-09-10

**Authors:** Sixiao Xin, Jingyi Hou, Liang Zou, Zhiyun Han, Zhen Li, Hanwen Ren

**Affiliations:** 1 School of Electrical Engineering, Shandong University, Jinan 250061, China; 202420765@mail.sdu.edu.cn (S.X.); hanzhiyun@sdu.edu.cn (Z.H.); lizzz1105@163.com (Z.L.); 2 State Grid Huzhou Electric Power Supply Company, Huzhou 313099, China; houjingyi0119@163.com; 3 State Key Laboratory of Alternate Electrical Power System with Renewable Energy Sources, North China Electric Power University, Beijing 102206, China; rhwncepu@ncepu.edu.cn

**Keywords:** epoxy resin, molecular dynamics simulation, nanofiller, thermal diffusion coefficients, glass transition temperature, dielectric properties

## Abstract

In high-humidity environments, the epoxy resin solid insulation materials of high-frequency transformers are prone to aging, resulting in varying degrees of deterioration in the material’s dielectric properties and other aspects. To enhance the adaptability of epoxy resin in high humidity environments, this paper, based on the molecular dynamics simulation method, establishes epoxy resin-based nanocomposites with doped nanofillers: a pure epoxy resin model and three epoxy resin models, respectively, doped with carbon nanotubes, graphene(GR), and SiO_2_. Based on the above models, using LAMMPS-17Apr2024, the thermal diffusion coefficients (thermal conductivity and specific heat capacity), glass transition temperatures, and dielectric constants under different moisture contents are calculated. The results show that the various properties of the epoxy resin nanocomposites doped with nanofillers have been improved to varying degrees. Among them, the GR/epoxy resin composite model shows the most significant improvements in thermal conductivity, thermal diffusivity, and glass transition temperature, and the SiO_2_/epoxy resin composite model has the best dielectric properties. Considering the high-temperature operation conditions and heat dissipation requirements of the high-frequency transformer, the GR-enhanced epoxy resin becomes the optimal filler choice.

## 1. Introduction

In the fields of AC/DC hybrid distribution networks and large-scale distributed renewable energy power generation systems, high-capacity, high-frequency transformers are core power equipment [1,2,3]. The epoxy solid-sealed high-frequency transformer uses epoxy resin solid insulating materials to achieve overall insulation of the equipment, which can effectively reduce insulation distance, reduce equipment volume, and improve power density [4,5]. However, high humidity environments may cause the aging of epoxy resin, leading to varying degrees of deterioration in material dielectric properties and thus reducing the service life. Therefore, it is urgent to deeply study the change laws of the performance parameters of epoxy resin under water intrusion and propose corresponding modification and enhancement methods to ensure the long-term stable operation of electrical equipment represented by high-frequency transformers in high humidity environments.

Domestic and foreign scholars have conducted in-depth research on the aging of epoxy resins due to moisture absorption. Li Guangmao et al. conducted a wet-heat aging experiment on epoxy resins, studying the water absorption characteristics and failure mechanism of epoxy resins in a humid and hot environment [6]. Li Yafeng et al. conducted a wet-heat aging test on epoxy resin-cured samples, exploring the influence of aging temperature on its dielectric properties and analyzing the mechanism of wet-heat aging [7]. However, current research mainly focuses on the macroscopic analysis of the wet-heat aging of epoxy resins, while there is relatively less research on the failure mechanism of epoxy resin aging due to moisture absorption at the microscopic scale.

Reducing the equilibrium water absorption rate of epoxy resin can effectively slow down the penetration of water molecules and the damage to the material, which can be achieved through two main methods [8]. One is to modify the chemical structure of the epoxy resin to reduce its hydrophilicity, including increasing the crosslinking degree of the epoxy resin and using different types of monomers; the other method is to add other substances, such as functional fillers, modifiers, etc. Using functional fillers not only provides an additional barrier to the structure but also improves the overall performance of the epoxy resin. Hadi Khoramishad added Fe_2_O_3_ nanoparticles and graphene oxide nanoplatelets to the epoxy resin, and the nano-fillers reduced the water absorption rate by restricting the movement of water within the epoxy resin, while also improving the fracture strength of the material [9]. Starkova, O studied the thermal mechanical properties of epoxy resin samples filled with thermally reduced graphene oxide and multi-wall carbon nanotubes, and found that the water content of the epoxy resin, containing 0.3 wt% thermally reduced graphene oxide and multi-wall carbon nanotubes, was three times lower than that of pure epoxy resin, and the thermal mechanical properties decreased more slowly [10]. Amirbek Bekeshev modified the amino groups on the surface of aluminum nitride through chemical methods, dispersed the aluminum nitride with amino groups into the epoxy resin composition, and found that the epoxy composite material prepared from aluminum nitride treated with amino acid had the best strength characteristics [11]. Zhang Jiacheng mixed fluorinated graphene with epoxy resin to prepare F-graphene/epoxy resin composite materials, this composite material exhibited high breakdown strength, high dielectric constant, low dielectric loss, low AC conductivity, and high thermal conductivity [12]. The above studies mainly focus on the changes in the water absorption rate and mechanical properties of the epoxy resin, and less attention is paid to the influence on thermal performance parameters.

With the improvement of computer computing power, molecular dynamic (MD) simulation has provided an effective means for studying the changes in the physical properties of matrix materials and the influence of nano-doped particles [13,14,15]. C. Baudot et al. conducted an experimental study on the covalent functionalization of carbon nanotube fillers and the covalent bonding with epoxy groups, ensuring the improvement of heat transfer [16]. Ding Mi et al. explored the influence of different functionalized carbon nanotube doping on the thermodynamic properties of epoxy resin/carbon nanotube composites based on molecular dynamic simulation [17]. Shen Lin used the molecular dynamics simulation method to design epoxy-based nanocomposites and established a composite model of epoxy resin and four graphene nanosheets [18].

Under high-frequency operating conditions, the loss of the high-frequency transformer increases sharply. In addition to paying attention to the insulation performance of the insulating material itself, its thermal performance also needs to be considered. For an external environment with high humidity, the invasion of moisture will affect the electric field distribution of the insulating material, distort the electric field, and cause the accumulation of surface charges. Therefore, the change in its dielectric performance needs to be paid attention to. Based on MD simulation, this paper studies the influence of moisture invasion on the performance degradation of epoxy resin from a microscopic perspective and proposes corresponding modification enhancement methods. Firstly, pure cross-linked epoxy resin and epoxy resins doped with carbon nanotubes, graphene, and SiO_2_ are constructed as models. Secondly, based on LAMMPS, MD simulation is conducted for the aforementioned models, and the thermal diffusion coefficient (thermal conductivity and specific heat capacity), glass transition temperature, and dielectric constant under different moisture contents are calculated. Finally, the improvement effect of different nano-fillers’ doping on the performance of epoxy resin is discussed.

## 2. Construction of Epoxy Resin/Nano-Filler Doping Modification Model

### 2.1. Construction of Epoxy Resin Models Under Different Moisture Invasions

**Figure 1:**
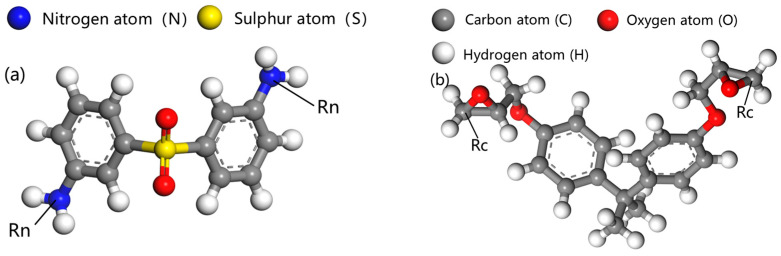
33DDS and DGEBA after marking the reaction site (**a**) 33DDS and (**b**) DGEBA.

The bisphenol A type epoxy resin is selected with the monomer and curing agent being bisphenol A diglycidyl ether (DGEBA) and 3,3′-diaminodiphenylsulfone (33DDS). The molecular models of these two components are constructed based on their molecular structures; the atoms involved in the cross-linking reaction are labeled; the reactive nitrogen atoms in 33DDS are marked as Rn, and the reactive carbon atoms in DGEBA are marked as Rc, as shown in Figure 1.

**Figure 2:**
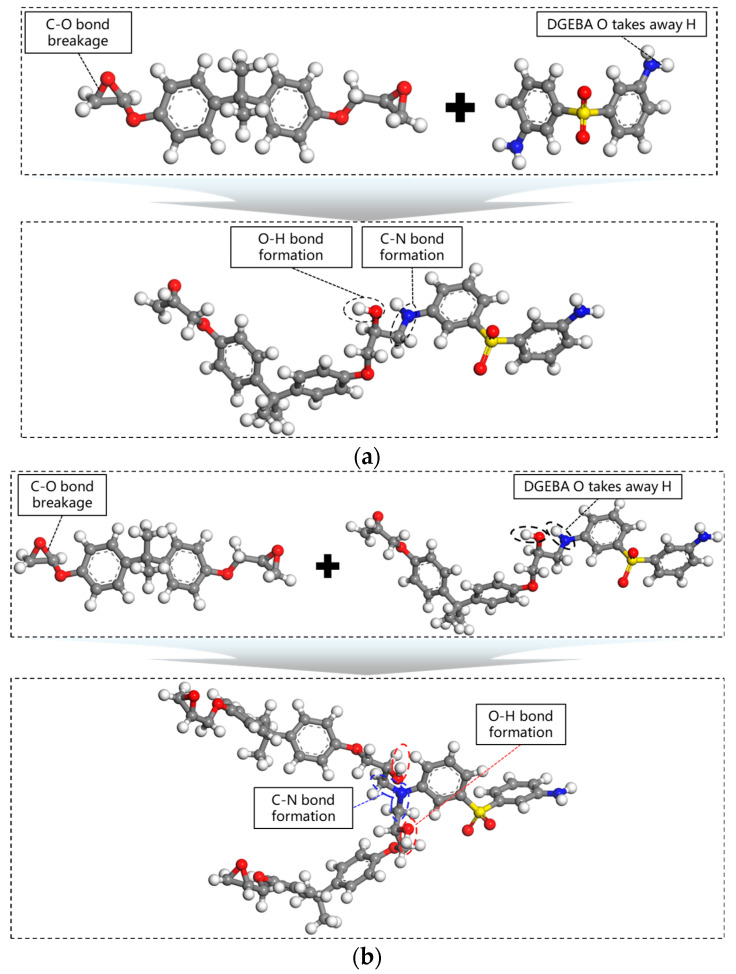
Schematic diagram of the cross-linking reaction mechanism (**a**) The first reaction of DGEBA with the -NH_2_ group; (**b**) The secondary reaction of DGEBA with the -NH group.

33DDS achieves crosslinking through a connection or condensation reaction between the amino group (-NH_2_) and the epoxy group [-CH(O)CH-] of DGEBA. The chemical reaction stage involves the ring-opening addition of primary amines with the epoxy groups and the catalytic action of hydroxyl groups. Among them, the open-loop addition is the main reaction, and its reaction mechanism consists of two steps, as shown in Figure 2. Each DGEBA has two -CH(O)CH- groups, and each 33DDS has two -NH_2_ groups. If one -NH_2_ reacts with two -CH(O)CH- groups, then the ratio of DGEBA to 33DDS is 2:1. Thus, a cross-linked epoxy resin model is constructed.

**Figure 3:**
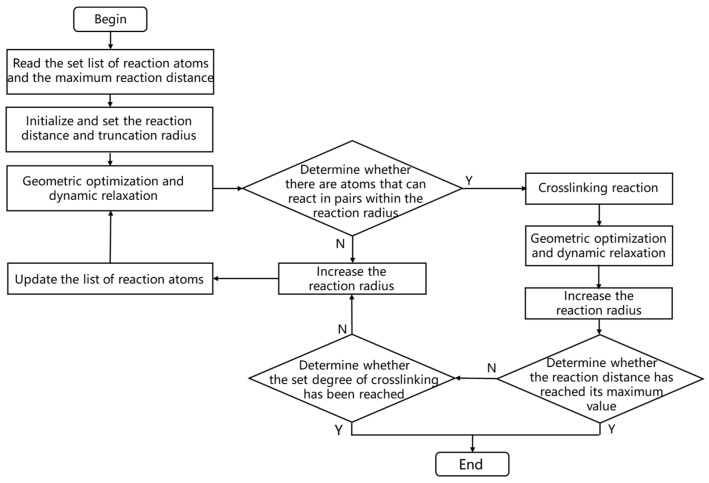
Cross-linking process flow.

**Figure 4:**
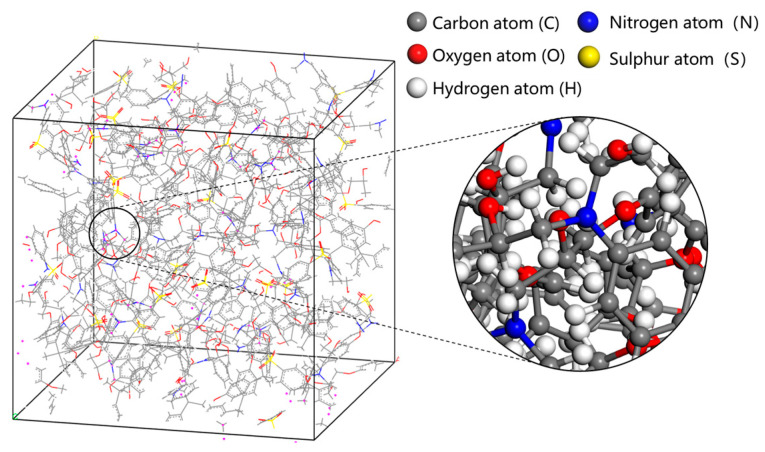
3D model of epoxy resin.

Fifty DGEBA and twenty-five 33DDS are placed in a periodic simulation box. The crosslinking process is shown in Figure 3. By reading in the list of reaction atoms, setting the maximum reaction distance and the crosslinking degree, the crosslinking reaction process is carried out at 1 atm (1 atm = 1.01 × 10^5^ Pa) and 500 K [19]. Subsequently, under the isothermal-isobaric system (NPT) (constant atomic number, constant pressure, and constant temperature), with a pressure of 1 atm and a temperature of 300 K set, 100 ps molecular dynamics optimization is performed on the cross-linked model to obtain a neat cross-linked epoxy resin (EP/neat) close to the real material, as shown in Figure 4. The entire simulation process uses the COMPASSII force field to control the intramolecular and intermolecular forces of the molecular model behavior [20].

**Figure 5:**
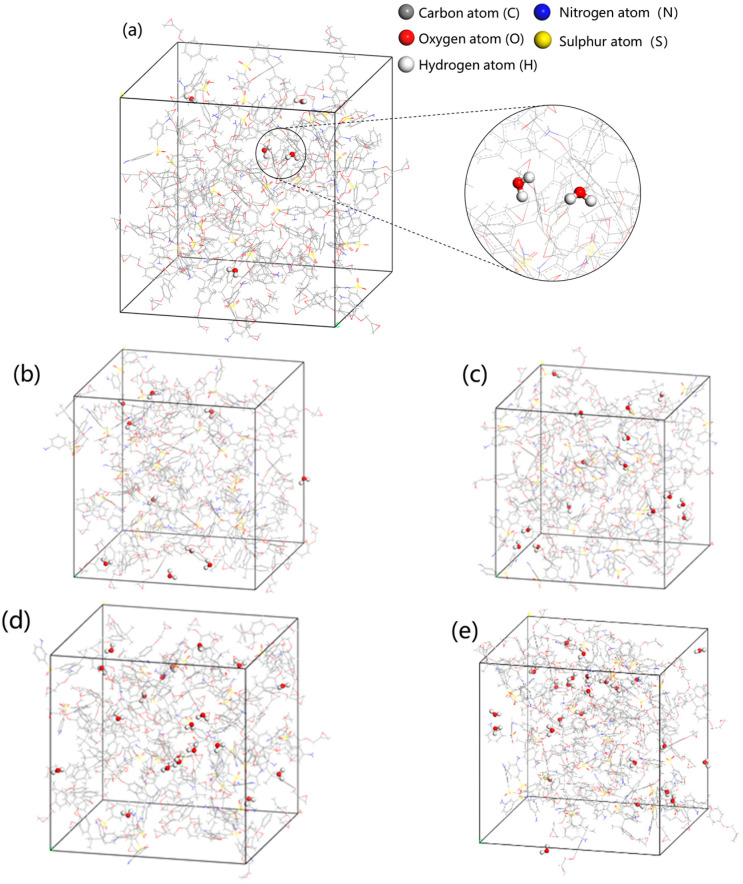
Five different epoxy resin models of moisture invasion: (**a**) EP/0.39 wt%H_2_O; (**b**) EP/0.77 wt%H_2_O; (**c**) EP/1.15 wt%H_2_O; (**d**) EP/1.53 wt%H_2_O; and (**e**) EP/1.95 wt%H_2_O.

Based on Figures 4, 5, 10, 15, 20, and 25, structurally optimized water molecules are added, respectively. The corresponding moisture absorption rates of the epoxy resin are 0.39 wt%, 0.77 wt%, 1.15 wt%, 1.53 wt%, and 1.90 wt%, among which the moisture absorption rate of 1.90 wt% is close to the saturated state [21]. Five epoxy resin composite models with different moisture absorption rates are finally constructed, as shown in Figure 5.

### 2.2. Construction of Epoxy Resin Nano-Doping Model

**Figure 6:**
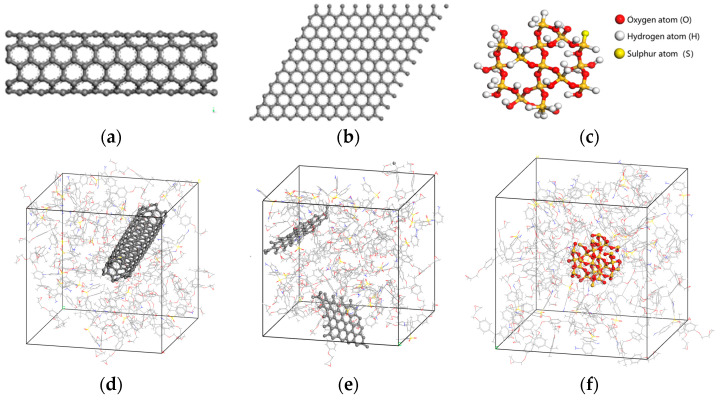
Nano-fillers and the corresponding nano-doping model structure of epoxy resin (**a**) SWCNT structure, (**b**) GR structure, (**c**) SiO_2_ structure, (**d**) EP/SWCNT structure, (**e**) EP/GR structure, and (**f**) EP/SiO_2_ structure.

Single-walled carbon nanotubes (SWCNT) [22], graphene (GR) [23], and SiO_2_ [24] are selected as nanofillers. The single-walled carbon nanotube/epoxy resin composite model (EP/SWCNT), graphene/epoxy resin composite model (EP/GR), and SiO_2_/epoxy resin composite model (EP/SiO_2_) are, respectively, constructed in the Materials Studio (MS) software. Meanwhile, different concentrations of water molecules are added to each composite model, respectively, to calculate various physical properties under different doping conditions. To ensure that the mass fraction difference in water molecules in the three types of nano-fillers is as small as possible at the same water absorption concentration after doping, the size and doping amount of the nano-fillers are adjusted to control the mass fraction of the nano-fillers to be basically the same. The structures of each nano-filler and the corresponding epoxy resin nano-doping model are shown in Figure 6.

## 3. Influence of Nano-Filler Doping Modification on the Physical Properties of Epoxy Resin Under Moisture Invasion

### 3.1. Modification and Improvement of the Thermal Diffusion Coefficient

The thermal diffusion coefficient represents the rate at which the temperature disturbance at one point of an object is transferred to another point, directly demonstrating the speed of heat conduction. Moreover, the larger the value, the better the heat transfer performance. The calculation formula is as follows [25]:

(1) $$a=\frac{\lambda }{\rho c}$$

where a is the thermal diffusion coefficient, λ is the thermal conductivity, ρ is the density, and c is specific heat capacity.

#### 3.1.1. Thermal Conductivity Calculation

The thermal conductivity is calculated by using the Nonequilibrium Molecular Dynamics (NEMD) [26] method based on Fourier’s law, similar to the experiment, and the local thermal bath method [27] is selected to generate the temperature gradient required for NEMD.

**Figure 7:**
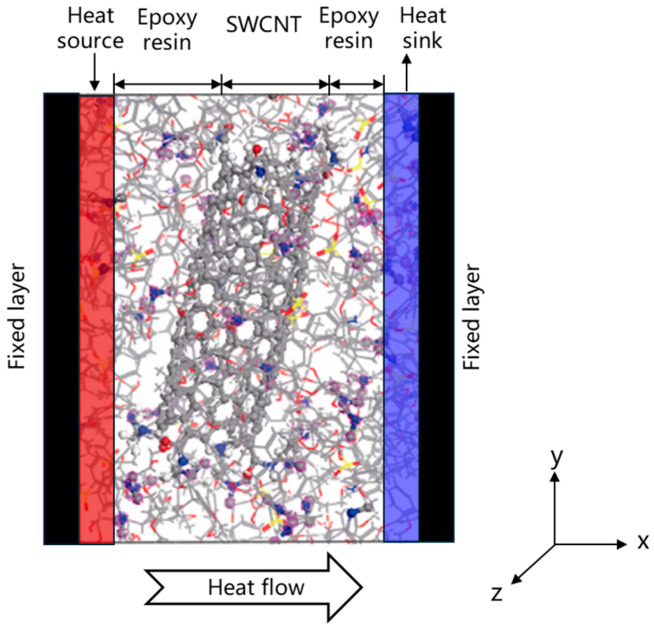
Schematic diagram of NEMD.

**Figure 8:**
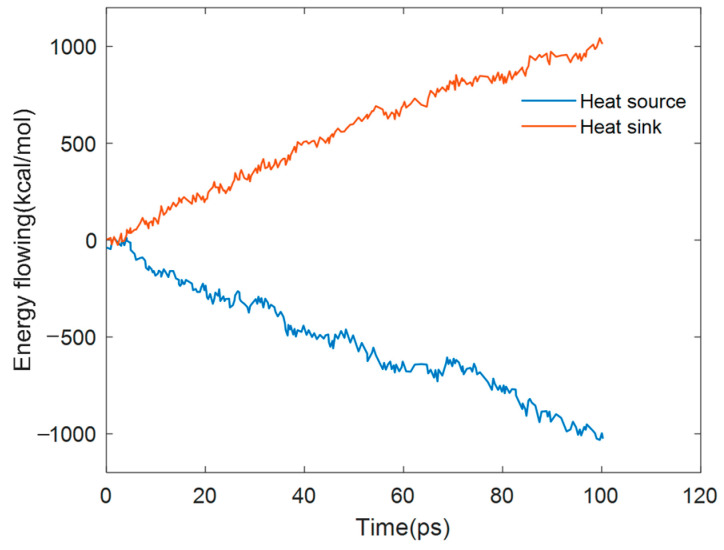
Variation in energy flowing into heat source and heat sink over time.

**Figure 9:**
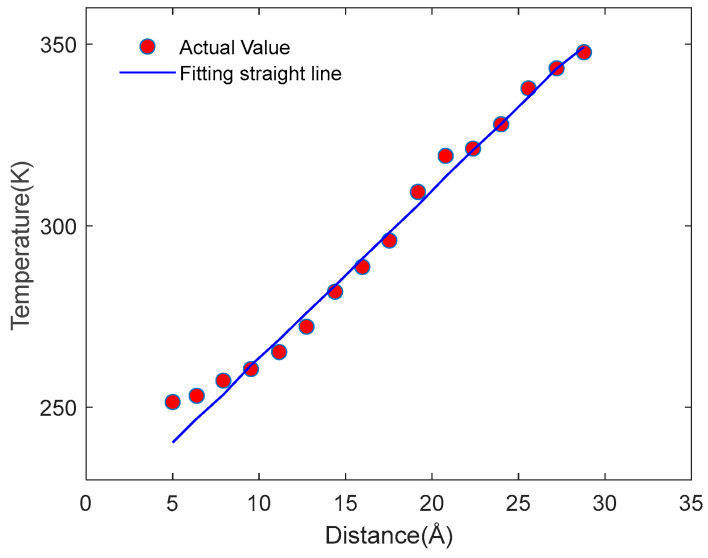
Temperature distribution after the heat flow stabilizes.

Based on LAMMPS [28], the model is first heated, as a whole, for 80 ps at 300 K under the canonical ensemble (NVT). The entire system is divided into 18 regions along the x direction, and then local heating at 100 ps is carried out under the micro-canonical ensemble (NVE). Fix the boundaries on both sides of the x-direction system as the fixed layers. The two areas closely adjacent to the fixed layer are, respectively, used as the heat source and heat sink, with temperatures set at 350 K and 250 K, respectively. The schematic diagram of the NEMD model is shown in Figure 7. Through the energy exchange from the heat source to the heat sink, a balanced and stable temperature gradient of energy is achieved. The variation in the energy flowing into the heat source and heat sink with the simulation time is shown in Figure 8, and the temperature gradient distribution at the steady state is shown in Figure 9. The thermal conductivity in the x direction can be obtained according to Fourier’s law:

(2) $$\kappa =\frac{Q}{\left|\frac{\delta T}{\delta x}\right|}$$

where Q is an unbalanced heat flow, defined as the energy passing through a given area perpendicular to the direction of the heat flow within a given time, and δT/δx represents the temperature gradient.

**Figure 10:**
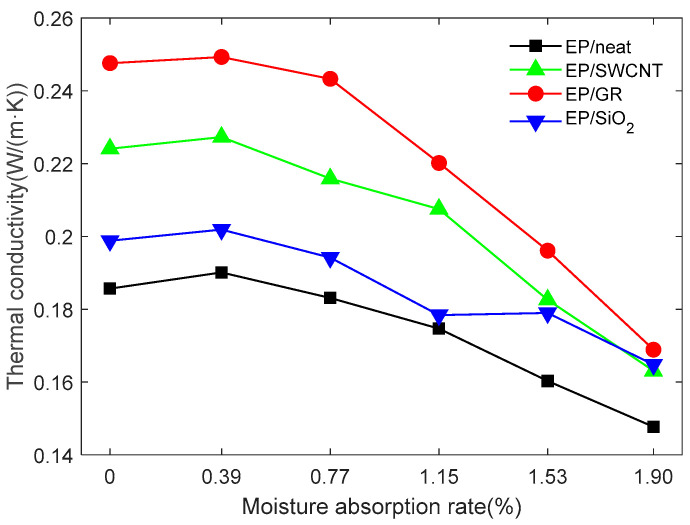
Variation curve of thermal conductivity of epoxy resin with moisture absorption rate after doping with different nano-fillers.

The x, y, and z directions of the epoxy resin models with different moisture absorption rates, doped and undoped, with three types of nano-fillers are repeated five times, respectively, and the average of the five calculation results is taken as the final thermal conductivity calculation result. The overall thermal conductivity is taken as the arithmetic mean of the thermal conductivities in the x, y, and z directions, and the result is shown in Figure 10.

When no nano-fillers are doped, the thermal conductivity of epoxy resin in the absence of moisture is 0.1857 W/(m*K), which is close to the experimentally measured data of 0.19 W/(m*K) [29]. With the increase in moisture absorption rate, the thermal conductivity of epoxy resin initially increases slightly, then gradually decreases, and significantly drops when there is high moisture intrusion. At the initial stage of water intrusion, a small amount of water molecules fill the free volume inside the epoxy resin, thereby improving the continuity of the local thermal conductivity path and reducing the thermal resistance of the material. When an equal amount of water invades, water molecules combine with polar groups of epoxy resin (such as hydroxyl groups or ether bonds), forming intermolecular hydrogen bonds, which disrupt the original cross-linked network of epoxy resin and reduce the thermal conductivity of the material. When the amount of water intrusion is large, the microscopic interior of the epoxy resin is completely filled with water molecules, and even a continuous water network is formed. The heat conduction channels of the epoxy resin are damaged or blocked. At the same time, high water intrusion will cause the epoxy resin to expand, thereby further increasing the intermolecular distance, weakening the energy transfer between chain segments, and reducing the total thermal conductivity.

There are significant differences in the improvement effect on the thermal conductivity of epoxy resin and the attenuation trend after water absorption by different nano-doped materials. Under waterless conditions, EP/GR exhibits the highest overall thermal conductivity of 0.2476 W/(m*K), which is superior to EP/SWCNT of 0.2241 W/(m*K) and EP/SiO_2_ of 0.1988 W/(m*K), but both are higher than the undoped epoxy resin. Under low humidity conditions, the overall thermal conductivity of EP/GR slightly increases to 0.2493 W/(m*K), while the thermal conductivities of EP/SWCNT and EP/SiO_2_ only fluctuate slightly. Under medium and high humidity conditions, the thermal resistance of EP/GR increases due to the accumulation of moisture between the lamellar layers, eventually dropping to 0.1689 W/(m*K). The thermal conductivity of EP/SWCNT decreases significantly due to the interference of interface scattering on its one-dimensional heat conduction path, eventually dropping to 0.1631 W/(m*K). The thermal conductivity of EP/SiO_2_ decreases the least under high humidity and heat conditions, from 0.1988 W/(m*K) to 0.1648 W/(m*K).

In epoxy resin composites, GR exhibits the best thermal conductivity due to its two-dimensional layered structure and the high in-plane thermal conductivity of the sp^2^-hybrid carbon layers. Its hydrophobic surface and tightly stacked layered structure effectively prevent water molecules from penetrating, maintaining stable van der Waals forces and π-π stacking interface coupling, thereby reducing the interface thermal resistance. In contrast, the curved surface of SWCNT increases phonon scattering, and its tube gaps may allow water molecules to penetrate it, further disrupting the thermal conductive network. SiO_2_ not only has a low intrinsic thermal conductivity but, also, its hydrophilic surface forms a hydration layer under humidity, significantly increasing the interface thermal resistance through hydrogen bond competition. Therefore, the layered structure of GR, at the molecular level, through efficient phonon transmission and anti-wetting interface interactions, enables EP/GR to maintain the optimal thermal conductivity performance under different humidity conditions.

#### 3.1.2. Specific Heat Capacity Calculation

Specific heat capacity refers to the amount of heat absorbed or released by a unit mass of a certain substance when its temperature rises or drops by a unit, which is calculated using the energy fluctuation method [30]. In the calculation, it is assumed that all atoms are excited, so quantum correction is required. The formula for calculating the specific heat capacity after correction is as follows [31]:

(3) $$c=k_{c}\frac{\langle E^{2}\rangle -{\langle E\rangle }^{2}}{k_{B}T^{2}}$$

where $k_{c}$ is the correction factor, E is the sum of the total potential energy and kinetic energy, T is the system temperature, and $k_{B}$ is the Boltzmann constant, approximately 1.38 × 10^−23^ J/K.

**Figure 11:**
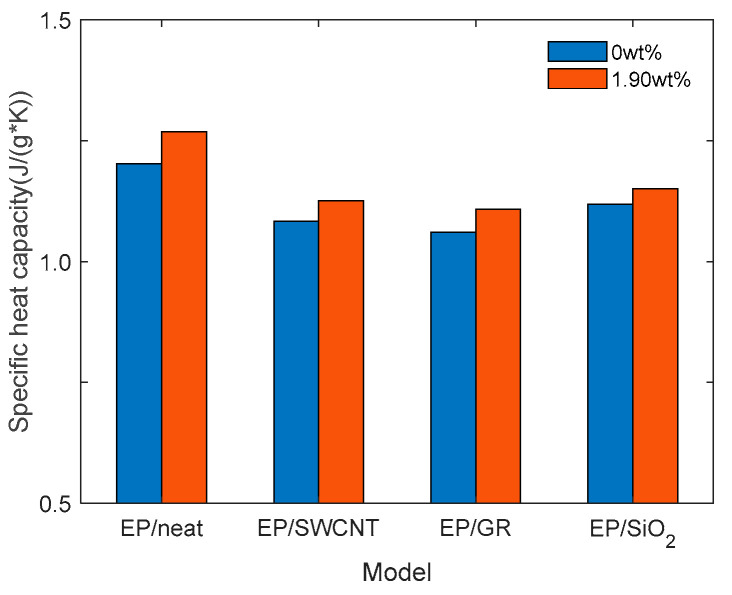
Specific heat capacities of the four models before and after saturated water absorption.

To simplify the calculation process, the dry state (0 wt%) model and the saturated water absorption concentration (1.90 wt%) model of the pure epoxy resin model and the epoxy resin model doped with three kinds of nano-fillers are taken to calculate the specific heat capacity. Each group of epoxy resin models is operated at a temperature of 300 K for 100 ps under NVT. Five repeated calculations are conducted for each group of models, and the average value is taken as the final calculation result of specific heat capacity. The results are shown in Figure 11.

The specific heat capacity of EP/neat in the dry state is 1.203 J/(g*K), which is similar to the specific heat capacity of epoxy resin, measured experimentally in reference [32], at 1.235 J/(g*K). After doping with the three kinds of nano-fillers, each specific heat capacity decreases. This is because the specific heat capacities of SWCNT, GR, and SiO_2_ are all lower than that of the epoxy resin matrix. After doping, the overall specific heat capacity of the system is the weighted average of the matrix and the nano-fillers. Due to the lower specific heat capacity of the fillers, it pulls down the overall specific heat capacity. From the perspective of the changes in specific heat capacity before and after saturated water absorption, the doping of nano-fillers reduces the growth rate of specific heat capacity. Its high thermal conductivity accelerates the uniform distribution of heat and reduces the dynamic heat absorption contribution of complex chain segments, which is macroscopically manifested as a decrease in specific heat capacity.

The thermal diffusivity is related to thermal conductivity, specific heat capacity, and density. According to Equation (1), the thermal diffusivity of four models can be obtained, as shown in Table 1. Compared with EP/neat, in the dry state, the thermal diffusion coefficients of EP/SWCNT, EP/GR, and EP/SiO_2_ increase by 17.74%, 45.03%, and 7.80%, respectively. Under saturated conditions, they increase by 17.24%, 21.35%, and 11.26%, respectively. Among them, EP/SWCNT and EP/GR have significant advantages in improving thermal performance.

### 3.2. Modification and Improvement of Glass Transition Temperature

In this section, the mean squared displacement (MSD) method of cross-linked nitrogen atoms is adopted to determine the glass transition temperatures of the four models [33], and the results are shown in Table 2.

In the dry state, the glass transition temperature of EP/neat is 480.34 K, which is close to the result measured in reference [34]. Under a dry environment, compared with EP/neat, the glass transition temperatures of EP/SWCNT, EP/GR, and EP/SiO_2_ increase by 13.62 K, 49.78 K, and 9.75 K, respectively. Under the saturated state, the glass transition temperatures of EP/SWCNT, EP/GR, and EP/SiO_2_ increase by 17.05 K, 66.22 K, and 15.70 K, respectively, compared with EP/neat. All three fillers can, to some extent, suppress the drop in glass transition temperature during the moisture absorption process, mainly because they form an effective physical barrier in the epoxy resin matrix. Among them, GR has the most significant effect, followed by SWCNT. Due to its large planar structure, GR can form extensive π-π stacking and van der Waals interactions with epoxy resin, significantly restricting the chain segment movement of the polymer. Although the one-dimensional tubular structure of SWCNT can also limit the chain segment movement through similar interactions, it is limited by its smaller contact area, resulting in a less-enhanced effect. As a zero-dimensional particle, SiO_2_ mainly forms hydrogen bond interactions with epoxy resin through surface hydroxyl groups. However, this interaction is easily disrupted by water molecules in a humid environment, leading to the weakest effect on the glass transition temperature increase.

### 3.3. Modification and Improvement of Dielectric Performance

The static relative dielectric constant of the composite material is calculated based on the total dipole moment of the model and the standard dipole moment fluctuation formula [35].

(4) $$\varepsilon =1+\frac{1}{3Vk_{B}T\varepsilon_{0}}(\langle M^{2}\rangle -{\langle M\rangle }^{2})$$

where V is volume, M is the dipole moment for each step size, $\langle M^{2}\rangle$ represents the average value of the squares of the dipole moments, and ${\langle M\rangle }^{2}$ represents the square of the average dipole moment.

**Figure 12:**
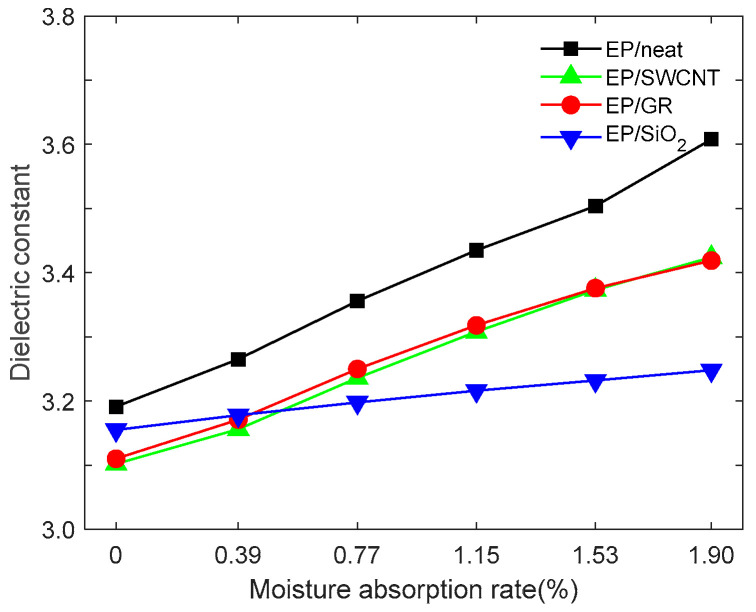
Dielectric constant of epoxy resin varies with the moisture absorption rate after doping with different nano-fillers.

Under NVT, all models are relaxed for 100 s at a temperature of 300 K to reach a stable state. Subsequently, in LAMMPS, 75 ps molecular dynamics simulation calculations are carried out under the NVT at 300 K, respectively, and the dipole moments of the entire system are obtained, respectively. The corresponding dielectric constants are calculated according to Formula (4). Each model is calculated five times, and the average value is taken as the dielectric constant value of the model, as shown in Figure 12.

The dielectric constant of EP/neat in the dry state is 3.191, which is close to the experimental result [36]. The dielectric constants of epoxy resins in different doping systems are all lower than those of pure epoxy resins, and they increase with the increase in moisture absorption rate, but there are significant differences in the extent of change. The dielectric constant of the EP/neat increases from 3.191 to 3.608, with an increase in approximately 13.1%, showing maximum moisture sensitivity. The increases in the EP/SWCNT and EP/GR are 10.4% and 9.9%, respectively, and the variation range is smaller than that of the undoped system. The dielectric constant of the EP/SiO_2_ model in a dry state is higher than that of EP/SWCNT and EP/GR, but its dielectric constant is almost unaffected by humidity, with an increase in only 2.9%. This is because the two-dimensional layered structure of GR effectively blocks the penetration of water molecules through its large area of hydrophobic surface and tight stacking property. At the same time, it forms stable π-π stacking and van der Waals interactions with epoxy resin, significantly inhibiting the influence of water molecules on the polarity of the polymer. Although the one-dimensional tubular structure of SWCNT can also limit the diffusion of water molecules through hydrophobicity and interfacial interactions, its relatively small contact area and potential tendency to aggregate result in a slightly inferior protective effect compared to GR. As a hydrophilic zero-dimensional particle, the surface hydroxyl groups of SiO_2_ can form hydrogen bond networks with epoxy resin. However, these polar groups will preferentially adsorb water molecules in a humid environment. Due to the fact that SiO_2_ is non-conductive and uniformly dispersed, its dielectric constant is the least sensitive to changes in humidity.

### 3.4. Comprehensive Performance Evaluation

Table 3 shows the specific performance comparison of different fillers (GR, SWCNT, SiO_2_, and undoped systems) in terms of moisture aging resistance. With the increase in moisture absorption rate, the reinforcing systems of different nano-fillers show significant differences in thermal diffusivity, glass transition temperature, and dielectric properties. Among them, GR has the best comprehensive performance, demonstrating the highest thermal diffusivity, the lowest drop in glass transition temperature, and relatively low humidity sensitivity, making it suitable for high-temperature and high-humidity environments. Next, SWCNT has a one-dimensional reinforced structure that significantly improves the strength and stability of epoxy resin under dry conditions, but its moisture sensitivity is slightly inferior to that of GR. It is suitable for neutral environments with certain requirements for thermal performance. SiO_2_ has a significant effect in inhibiting the deterioration of dielectric properties, but it has poor thermal diffusion performance and high moisture sensitivity, making it suitable for application scenarios with higher requirements for the stability of electrical performance. Based on the high-temperature operation requirements of high-frequency transformers, GR-reinforced epoxy resin has become the optimal filler choice.

## 4. Conclusions

In this paper, models of epoxy resin materials under different moisture absorption rates are established, and a modification and enhancement method of nano-doping is proposed. Based on MD simulation, the thermal properties of materials are calculated, including the thermal diffusion coefficient (thermal conductivity and specific heat capacity), glass transition temperature, and dielectric constant. The parameters of pure cross-linked epoxy resins with different water contents and nano-doped epoxy resin composites are compared to analyze the influence of nano-fillers on the physical properties of epoxy resin composites. The main conclusions are as follows:

(1) The thermal diffusion coefficients of epoxy resin nano-filler composites have all been improved. Before water absorption, the thermal diffusivity coefficient of EP/neat is the lowest, EP/GR is the highest, EP/SWCNT is second, and EP/SiO_2_ is slightly higher than EP/neat. After absorbing water, the thermal diffusion coefficients of all systems decrease, and the order of thermal diffusion coefficients of each model in the saturated state remains the same as before water absorption.

(2) The doping of nano-fillers all increase the glass transition temperature of the epoxy resin nanocomposite system. In a dry environment, compared with EP/neat, the glass transition temperatures of EP/SWCNT, EP/GR, and EP/SiO_2_ increase by 13.62 K, 49.78 K, and 9.75 K, respectively. In the saturated state, the glass transition temperatures of EP/SWCNT, EP/GR, and EP/SiO_2_ increase by 17.05 K, 66.22 K, and 15.70 K, respectively, compared with EP/neat.

(3) The addition of nano-fillers enhances the dielectric properties of epoxy resin. From a dry environment to a saturated state, the dielectric constant of EP/neat increases from 3.191 to 3.608, showing maximum moisture sensitivity. The growth rates of the EP/SWCNT and EP/GR systems are 10.4% and 9.9%, respectively, which are superior to EP/neat. The dielectric constant of EP/SiO_2_ increases the least under humidity changes, showing the lowest humidity sensitivity.

(4) A comprehensive comparison of the three nano-doped epoxy resin composite models shows that EP/GR exhibits the highest thermal diffusivity, the lowest drop in glass transition temperature, and relatively low moisture sensitivity. In the high-temperature operating environment of high-frequency transformers, GR-reinforced epoxy resin becomes the optimal filler choice.

This paper systematically investigates the influence mechanism of moisture absorption rate on the performance parameters of epoxy resins and their composite materials. In practical engineering applications, materials often simultaneously bear the combined effects of mechanical loads and water erosion. This multi-field coupling effect significantly accelerates the degradation of material performance. During the process from the initial drying state to the moisture absorption equilibrium, the material performance exhibits obvious time-dependent characteristics. Subsequent work can include the following: first, establishing a degradation model for material performance under mechanical–chemical coupling effects reveals the time-dependent laws of material performance during water absorption; second, exploring the influence of epoxy resin matrix doping (such as introducing thermal conductive fillers like boron nitride and aluminum nitride) on material properties. These studies will provide theoretical basis for the durability design and performance optimization of epoxy resin composites.

## Figures and Tables

**Table 1:** Thermal diffusion coefficients and growth rates of the four models before and after saturated water absorption.

Model	Thermal Diffusion Coefficient in 0 wt% State (m^2^/s)	Thermal Diffusion Coefficient Growth Rate in 0 wt% State (%)	Thermal Diffusion Coefficient in 1.90 wt% State (m^2^/s)	Thermal Diffusion Coefficient Growth Rate in 1.90 wt% State (%)
EP/neat	0.1308	-	0.1021	-
EP/SWCNT	0.1540	17.74	0.1197	17.24
EP/GR	0.1897	45.03	0.1239	21.35
EP/SiO_2_	0.1410	7.80	0.1136	11.26

**Table 2:** Glass transition temperatures of the four groups of models at different moisture absorption rates.

Moisture Absorption Rate/%	Glass Transition Temperature/K			
	EP/neat	EP/SWCNT	EP/GR	EP/SiO_2_
0	480.34	493.96	530.12	490.10
0.39	472.20	486.24	524.50	483.87
0.77	461.76	477.71	519.17	475.26
1.15	451.29	468.28	515.09	469.02
1.53	445.85	463.97	511.57	463.61
1.90	442.61	459.66	508.83	458.31

**Table 3:** Performance of each system before and after moisture exposure.

	EP/Neat	EP/GR	EP/SWCNT	EP/SiO_2_
Thermal diffusion coefficient.	Decrease significantly after becoming damp.	Strongest ability to maintain.	Better, slightly weaker in high humidity.	Worse.
Glass transition temperature.	Poor moisture tolerance.	Minimum decline rate.	Suboptimum.	General stability.
Dielectric performance.	Worst, with an increase in 13.1%.	Relatively low, with an increase in 9.9%.	Relatively low, with an increase in 10.4%.	Lowest, with an increase in 2.9%.

## Data Availability

The original contributions presented in this study are included in the article. Further inquiries can be directed to the corresponding author.
